# Submillisievert standard-pitch CT pulmonary angiography with ultra-low dose contrast media administration: A comparison to standard CT imaging

**DOI:** 10.1371/journal.pone.0186694

**Published:** 2017-10-18

**Authors:** Saravanabavaan Suntharalingam, Christian Mikat, Elena Stenzel, Youssef Erfanian, Axel Wetter, Thomas Schlosser, Michael Forsting, Kai Nassenstein

**Affiliations:** Department of Diagnostic and Interventional Radiology and Neuroradiology, University Hospital Essen, Essen, Germany; University of Tennessee Health Science Center, UNITED STATES

## Abstract

**Objectives:**

To evaluate the image quality and radiation dose of submillisievert standard-pitch CT pulmonary angiography (CTPA) with ultra-low dose contrast media administration in comparison to standard CTPA.

**Materials and methods:**

Hundred patients (56 females, 44 males, mean age 69.6±15.4 years; median BMI: 26.6, IQR: 5.9) with suspected pulmonary embolism were examined with two different protocols (n = 50 each, group A: 80 kVp, ref. mAs 115, 25 ml of contrast medium; group B: 100 kVp, ref. mAs 150, 60 ml of contrast medium) using a dual-source CT equipped with automated exposure control. Objective and subjective image qualities, radiation exposure as well as the frequency of pulmonary embolism were evaluated.

**Results:**

There was no significant difference in subjective image quality scores between two groups regarding pulmonary arteries (p = 0.776), whereby the interobserver agreement was excellent (group A: k = 0.9; group B k = 1.0). Objective image analysis revealed that signal intensities (SI), signal-to-noise ratio (SNR) and contrast-to-noise ratio (CNR) of the pulmonary arteries were equal or significantly higher in group B. There was no significant difference in the frequency of pulmonary embolism (p = 0.65). Using the low dose and low contrast media protocol resulted in a radiation dose reduction by 71.8% (2.4 vs. 0.7 mSv; p<0.001).

**Conclusions:**

This 80 kVp standard pitch CTPA protocol with 25 ml contrast agent volume can obtain sufficient image quality to exclude or diagnose pulmonary emboli while reducing radiation dose by approximately 71%.

## Introduction

Computed tomography pulmonary angiography (CTPA) is recommended as first-line imaging modality in patients with suspected pulmonary embolism (PE) due to its high diagnostic accuracy [1]. Demographic and medical developments creating more elderly, multimorbid and immobilized patients, as well as the broad acceptance and availability of CT have led to an increasing number of CTPA examinations [2], but pulmonary embolism is found only in the minority of cases (9–35%) [3, 4]. Therefore the increase in radiation exposure to patients related to CTPA has raised concerns [5].

In addition to the radiation exposure intrinsic to CT, the necessary intravenous contrast media injection required for CTPA is another important issue of interest. The intravenous application of iodinated contrast agent is problematic in patients with impaired renal function because of the risk of contrast medium induced nephropathy [6, 7]. Unfortunately, many patients at risk for pulmonary embolism are elderly and present with comorbid conditions that increase the risk for contrast medium induced nephropathy.

Besides clinical routine methods like iterative image reconstruction or tube current modulation techniques, different approaches reducing radiation exposure and/ or contrast media demand of CT angiography exist: Reduction of the tube voltage leads tosignificant reduction in radiation exposure, due to the fact that radiation exposure increases approximately with the square of the tube voltage [8]. Moreover, imaging at low tube voltages results in increased contrast conspicuity due to an approximation to the k-edge of iodine, which allows in turn a reduction of the contrast media dosage [9]. High-pitch imaging is another method to reduce the radiation exposure of CT angiography.

Recently some studies showed that a high pitch protocol with low tube voltage and reduced contrast media dosage is feasible to detect pulmonary embolism [10, 11]. However, high-pitch CTPA in combination with low contrast media volume does not allow a sufficient evaluation of the aorta [12]. However, aortic dissection (AD) especially of the ascending aorta is often an important differential diagnosis in patients with suspected pulmonary embolism that requires urgent diagnosis and treatment [13, 14].

To the best of our knowledge, none of the existing studies have analyzed so far whether a CTPA protocol combining low tube voltage, standard pitch and ultra-low contrast media administration is feasible to detect pulmonary emboli and to assess the ascending aorta sufficiently. Therefore, the aim of the study was to assess the image quality, radiation dose and diagnostic accuracy of submillisievert standard-pitch CTPA with ultra-low dose contrast media administration in comparison to standard CTPA.

## Material and methods

This retrospective single center study was approved by the University Hospital Essen ethics committee. Written informed consent was waived by the Institutional Review Board due to the retrospective character of the study and anonymized data evaluation.

### Patients

The study was approved by the local ethics committee. Fifty consecutive patients (group A: 31 females/ 19 males; mean age: 70.3 ± 16.1 years; body-mass-index: 25.9 kg/m², IQR 6.1 kg/m², range 22.8 to 28.9 kg/m²) were scanned with a submillisievert standard-pitch CTPA with ultra-low dose contrast media administration, and fifty consecutive patients (group B: 25 females/ 25 males; mean age: 68.9 ± 14.8 years; body-mass-index: 27.3 kg/m², IQR 5.7 kg/m², range 24.6 to 30.3 kg/m²) were examined with standard CTPA.

### Image acquisition

All examinations were performed on a second generation dual-source CT (Somatom Definition FLASH, Siemens Healthineers, Forchheim, Germany). A scout view covering the patient from the neck to the upper abdomen was acquired (tube voltage: 100 kV, tube current: 40 mA) in anteroposterior direction in supine position with elevated arms at suspended full inspiration, and the position for bolus tracking (level of the pulmonary trunk) as well as the scan range (lung apices to costodiaphragmatic recess) for CTPA was defined within the scout image. For bolus tracking a single slice covering the pulmonary trunk was repetitively acquired (tube voltage: 100 kV, tube current-time-product: 20 mAs, slice thickness: 10 mm, repetition time: 0.86 sec, start of the measurement: X sec after contrast injections), and a circular region-of-interest (ROI) of ~1 cm² was placed within the pulmonary trunk. In group A, 25 ml iobitridol with 350 mg iodine per ml (Xenetix 350, Guerbet, Roissy CdG Cedex, France) were injected in an antecubital vein via an indwelling venous cannula with a diameter of 18 G at a flow rate of 4 ml/sec followed by a chaser bolus of 30 ml physiologic salt solution at a flow rate of 4 ml/sec by an automated contrast injector (Medrad Stellant, Medrad Inc., Warrendale, Pennsylvania, USA), whereas in group B, 60 ml of the same contrast media were injected analogously. CTPA was acquired 5 seconds after reaching a threshold of 90 HU within the ROI at suspended full inspiration. For all CTPA examinations automated tube voltage selection (Care kV, Siemens Healthineers, Forchheim, Germany) was used with a quality reference tube voltage of 80 kV in group A and of 100 kV in group B, and the algorithm was set to the mode “dose saving optimized for contrast-enhanced angiography” (number 11 on the 12-point scale of CARE kV), resulting in the selection of 80 kV in all examinations of group A and of 100 kV in all examinations of group B. Automated tube current modulation (CARE Dose 4D, Siemens Healthineers, Forchheim, Germany) was used in all cases with a quality reference tube current-time product of 115 mAs in group A, and of 150 mAs in group B. In both groups, scans were acquired with 128x0.6 mm detector configuration, 76.8 mm beam collimation, a pitch of 1.2, and a tube rotation time of 0.28 sec (see Table 1). For image reconstruction Sinogram Affirmed Iterative Reconstruction (SAFIRE, Siemens Healthineers, Forchheim, Germany) was used with a moderate strength of 3 in group A and of 2 in group B (available strength of SAFIRE: 1 to 5, where a higher number implies a stronger noise reduction). Images were reconstructed in 1.5 mm slice thickness and 1.0 mm reconstruction interval using an I26s kernel.

**Table 1:** Scanning protocols of the submillisievert CTPA with ultra-low dose contrast media administration and the standard CTPA.

Parameter	Group A	Group B
Detector configuration (mm)	128x0.6	128x0.6
Pitch	1.2	1.2
Tube rotation time (sec)	0.28	0.28
Tube voltage (ref. kV)	80	100
Tube current (ref. mAs)	115	150
Contrast media volume	25	60

### Objective image quality assessment

Attenuation values were measured in one session by a radiologist with five years of experience by placing a region of interest (ROI) in the ascending aorta (AAO), the descending aorta (DAO), the main pulmonary artery (MPA), the right pulmonary artery (RPA), the left pulmonary artery (LPA) and peripheral pulmonary arteries, which included the right upper lobar, the right posterobasal segmental and its subsegmental branches on axial images. Furthermore, the mean signal intensity of the bilateral pulmonary lower lobes was measured at the aortic root level by averaging values of three measurements (ROI of ~2 cm^2^). In case of pre-existing pulmonary pathologies in these slices, measurements were performed in other lung lobes at the same slices. The signal intensity of the paraspinal muscle at the level of the pulmonary trunk was obtained by placing a ROI with an area of ~1 cm^2^. Background noise was measured in a circular ROI of ~2 cm^2^ placed in an artifact free region (air) 3 cm ventral of the thoracic wall. Signal-to-noise ratio (SNR) and contrast-to-noise ratio (CNR) between the target artery and paraspinal muscle were calculated.

### Subjective image quality assessment

All images were evaluated by two blinded readers (reader 1: 8 years of experience, reader 2: 5 years of experience) in random order. Overall image quality of the pulmonary arteries (main pulmonary arteries to the sub-segmental pulmonary arteries), the ascending aorta and the descending aorta were each subjectively scored on a three-point scale, as previously described [15]:

1. Good to excellent quality, meaning that enhancement allows confident diagnosis
2. Adequate quality, meaning that enhancement was inferior to those vessels scored as 1 but enabled the diagnosis with moderate confidence
3. Non-diagnostic quality, meaning that the images could not be used for diagnosis due to insufficient contrast enhancement.

### Detection of pulmonary emboli

The same blinded readers rated independently the presence of pulmonary embolism in a binary manner (pulmonary embolism vs. no pulmonary embolism).

### Estimation of radiation exposure

The volume CT dose index (CTDI_vol_), which was automatically calculated for a 32 cm body phantom by the CT scanner, and the dose-length-product (DLP) were recorded for each CT. The DLP was multiplied with a conversion factor of 0.014 mSv/mGy*cm to calculate an estimate of the effective dose (ED) [16].

### Statistical analysis

Statistical analysis was performed using MedCalc Version 13.3 (MedCalc Software, Mariakerke, Belgium). A D’Agostino-Pearson test was performed to test for normal distribution. Continuous data with normal distribution are given as mean ± standard deviation (SD), non-normally distributed data as median and interquartile range (IQR). Categorical data are expressed as absolute numbers. Mann-Whitney U test was performed as non-parametric test. Chi-square test was used to compare the frequency distribution of subjective image quality scores between the two groups. Kappa analysis was used to evaluate the inter-reader agreement. A p-value of ≤ 0.05 was considered statistically significant.

## Results

### Patients

There were no statistically significant differences between the two study groups in age (p = 0.661), body-mass-index (p = 0.08), or gender distribution (p = 0.234).

### Objective image quality assessment

Objective image quality analysis showed only by trend a slightly higher noise level in the images acquired with the submillisievert standard-pitch imaging approach; however, the difference in noise between both imaging approaches was marginal and did not reach any statistical significance. Overall slightly lower SI, SNR and CNR values were observed in the pulmonary artery tree in the submillisiervert standard-pitch CTPA with low-dose contrast media administration compared to standard CTPA (for details see Table 2). No differences in SI, SNR and CNR were observed in the ascending and descending aorta, in lung parenchyma as well as in the musculature between both imaging protocols (for details see Table 2).

**Table 2:** Objective image quality analysis in the two CTPA groups (group A: submillisievert standard-pitch CTPA with ultra-low dose contrast media administration, group B: standard CTPA).

		Group A		Group B		*p*
		Median	IQR	Median	IQR	
**Ascending aorta**	SI	201	157	189.5	101	0.4661
	SNR	9.4	8.5	9.2	7.1	0.8545
	CNR	7.6	8,2	7.1	6.1	0.6852
**Descending aorta**	SI	143	159	137.5	113	0.3
	SNR	7.2	7.6	6.1	6.4	0.65
	CNR	5.2	7.8	4.3	5	0.4
**Main pulmonary artery**	SI	269	140	343	138	0.0019
	SNR	12	4.1	15.5	8.9	<0.0001
	CNR	10.2	3,7	13	8.9	0.0001
**Left pulmonary artery**	SI	285	128	335.5	123	0.0277
	SNR	13.1	5.5	15	8	0.0011
	CNR	10.8	5.1	13.3	8.6	0.0018
**Right pulmonary artery**	SI	272	131	335.5	130	0.0844
	SNR	12.8	4.9	14.7	6.8	0.0031
	CNR	10.9	4.6	12.7	7.3	0.0063
**Lobar arteries**	SI	316.5	157	368.5	137	0.0765
	SNR	14.5	6.6	16.4	8.5	0.0057
	CNR	12.8	6	14.2	8.5	0.01
**Segmental arteries**	SI	292.5	131	344	136	0.0422
	SNR	13.1	6.4	15.8	7.4	0.0018
	CNR	10.6	6.3	13.8	6.9	0.0032
**Subsegmental arteries**	SI	290.5	117	344.5	120	0.1397
	SNR	13.4	6.4	15.8	8.2	0.0041
	CNR	11.4	5.8	13.6	8.8	0.0069
**Lung parenchyma**	SI	-788	76.5	-796	98	0.69
	SNR	-34.4	9.9	-38.2	13.3	0.23
**Muscle**	SI	38.5	19.0	43.5	17	0.3769
	SNR					
**Noise**		22.2	6.05	20.8	5.15	0.0523

*Abbreviations*: SI signal intensity; SNR signal-to-noise ratio, CNR contrast-to-noise ratio

### Subjective image quality assessment

**Fig 1:**
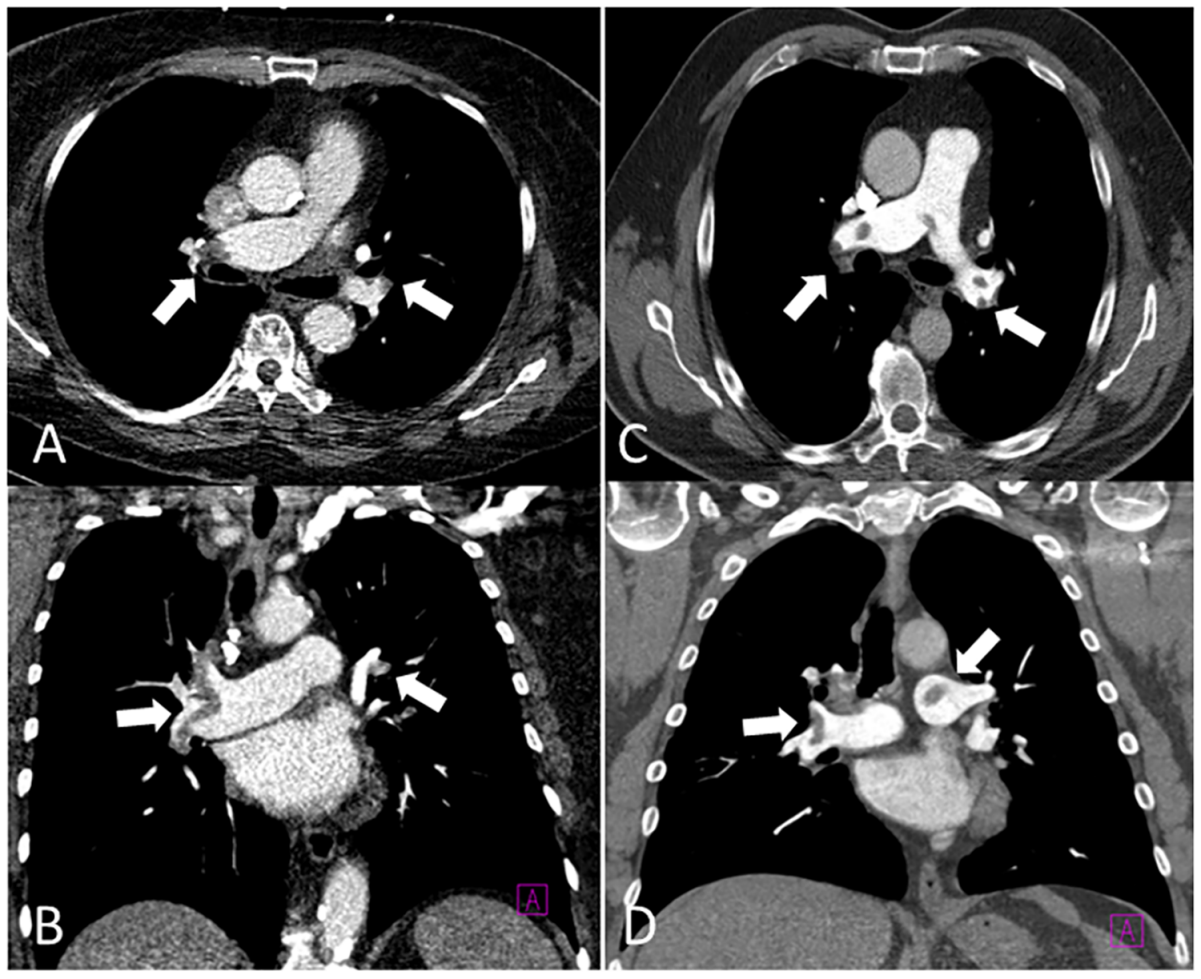
Axial and coronal CT images of the pulmonary arteries. On the left side (A + B) there is a CTPA acquired with 80 kVp and 25 ml contrast agent in a 55-year-old man with central and peripheral pulmonary emboli (see arrows). Additionally to the pulmonary arteries there is a sufficient opacification of the aorta. The CTPA on the right side (C + D) was acquired with 100 kVp and 60 ml contrast agent in a 55-year-old man with central and peripheral pulmonary emboli (see arrows). While the standard protocol shows a better image quality in terms of less image noise, the low dose and low contrast media protocol is feasible to detect a pulmonary embolism and pathologies in the aorta.

Subjective image quality assessment revealed a good to excellent quality (score 1) of the pulmonary artery tree in the vast majority of examinations performed with both CTPA approaches (Fig 1). However, the standard-pitch submillisievert CTPA protocol with ultra-low dose contrast media administration resulted by trend in a slightly higher number of examinations rated only as adequate image quality (score 2) compared to standard CTPA. Moreover, one examination performed with the standard-pitch submillisievert CTPA protocol was rated as non-diagnostic (score 3), whereas all standard CTPA examinations were categorized as diagnostic image quality (score 1 or 2). For details see Table 3.

**Table 3:** Subjective image quality evaluation of pulmonary arteries in the two study groups (group A: submillisievert standard-pitch CTPA with ultra-low dose contrast media administration, group B: standard CTPA).

	Quality score	Group A	Group B	*P*
Reader 1	1	39 (78%)	46 (92%)	0.776
	2	11 (22%)	3 (6%)	
	3	0 (0%)	1 (2%)	
Reader 2	1	38 (76%)	46 (92%)	0.795
	2	12 (24%)	3 (6%)	
	3	0 (0%)	1 (2%)	
Consensus reading	1	39 (78%)	46 (92%)	0.776
	2	11 (22%)	3 (6%)	
	3	0 (0%)	1 (2%)	
*Inter-rater agreement*		0.9	1.0	

Quality score 1: Good to excellent; quality score 2: adequate; quality score 3: non-diagnostic image quality

Concerning the ascending aorta, subjective image quality assessment showed diagnostic image quality (score 1 or 2) in the majority of cases with both imaging protocols. However, a non-diagnostic image quality of the ascending aorta was observed in 18% of the standard-pitch submillisievert CTPA examinations, and even in 14% of the standard CTPA examinations. Concerning the descending aorta, standard-pitch submillisievert CTPA resulted in a diagnostic image quality in 56% of cases, while standard CTPA showed a diagnostic image quality in 70% of cases. For details see Tables 4 and 5.

**Table 4:** Subjective image quality evaluation of the ascending aorta in the two study groups (group A: submillisievert standard-pitch CTPA with ultra-low dose contrast media administration, group B: standard CTPA).

	Quality score	Group A	Group B	*P*
Reader 1	1	22 (44%)	18 (36%)	0.144
	2	19 (38%)	25 (50%)	
	3	9 (18%)	7 (14%)	
Reader 2	1	22 (44%)	18 (36%)	0.082
	2	16 (32%)	25 (50%)	
	3	12 (24%)	7 (14%)	
Consensus reading	1	22 (44%)	18 (36%)	0.144
	2	19 (38%)	25 (50%)	
	3	9 (18%)	7 (14%)	
*Inter-rater agreement*		0.927	0.943	

Quality score 1: Good to excellent; quality score 2: adequate; quality score 3: non-diagnostic image quality

**Table 5:** Subjective image quality evaluation of the descending aorta in the two study groups (group A: submillisievert standard-pitch CTPA with ultra-low dose contrast media administration, group B: standard CTPA).

	Quality score	Group A	Group B	*P*
Reader 1	1	16 (32%)	14 (28%)	0.040
	2	12 (24%)	22 (44%)	
	3	22 (44%)	14 (28%)	
Reader 2	1	16 (32%)	14 (28%)	0.080
	2	12 (24%)	21 (42%)	
	3	22 (44%)	15 (30%)	
Consensus reading	1	16 (32%)	14 (28%)	0.080
	2	12 (24%)	21 (42%)	
	3	22 (44%)	15 (30%)	
*Inter-rater agreement*		1.0	0.975	

Quality score 1: Good to excellent; quality score 2: adequate; quality score 3: non-diagnostic image quality

### Detection of pulmonary emboli

Pulmonary embolism was found in a similar frequency with both imaging approaches (group A: 10 (20%) cases; group B: 14 (28%) cases; p = 0.65). The interobserver agreement for the diagnosis of pulmonary embolism was excellent (group A: k = 0.883; group B: k = 0.947).

### Radiation exposure

Based on CTDIvol, DLP and effective dose the radiation exposure was significantly lower in group A (all p <0.0001). Compared to group B the effective dose was 71% lower in group A. For details see Table 6.

**Table 6:** Radiation dose estimation for both CTPA approaches (group A: submillisievert standard-pitch CTPA with ultra-low dose contrast media administration, group B: standard CTPA).

	Group A		Group B		*p*
	Median	IQR	Median	IQR	
CTDI vol [mGy]	1.7	0.7	4.8	1.1	<0.0001
DLP [mGy*cm]	47.5	19.0	172.0	48.0	<0.0001
Effective dose [mSv]	0.7	0.3	2.4	0.7	<0.0001

## Discussion

The present study shows that our standard-pitch submillisievert CTPA with ultra-low contrast media administration allows the assessment of the pulmonary arteries as well as the ascending aorta comparably to standard CTPA with a 71% reduction in radiation exposure and a 60% reduction in contrast agent volume. To the best of the authors’ knowledge, the implemented CTPA protocol, which was not limited in regard to the patients’ weight or body-mass-index, featured the lowest radiation dose and contrast media dosage published so far for standard-pitch CTPA.

Several studies investigated low kVp protocols for CTPA: Wichmann et al compared a 70 kVp standard pitch protocol with standard CTPA acquired with 100 kVp using 70 ml contrast media administration in both protocols. They reported that compared to standard CTPA the low tube voltage CTPA protocol allows for significant radiation dose savings (0.95 ± 0.17 mSv vs. 2.3 ± 0.7 Sv) while maintaining a comparable objective image quality in terms of SNR and CNR [17]. Boos et al. tested a 70 kV low-pitch CTPA protocol with 40 ml contrast media application in comparison to a high-pitch protocol acquired with a reference tube voltage of 120 kV with 70 ml contrast media application, and found a significant reduction in radiation dose (2.0 ± 0.6 mSv vs. 3.9 ± 1.1 mSv) when using the 70 kV scanning protocol while maintaining a comparable image quality [18]. Compared to these recent low tube voltage standard-pitch CTPA studies, our scan protocol showed a further reduction in radiation dose (median dose: 0.7 mSv) and contrast media dosage while preserving a sufficient image quality.

Because high-pitch imaging is another method to further reduce radiation exposure of CT imaging, recent studies evaluated high pitch CTPA in combination with low tube voltage imaging for detection of pulmonary embolism: Li et al tested a high-pitch 70 kVp CTPA protocol with 40 ml contrast media in comparison to standard CTPA with 100 kVp and 60 ml contrast media and found a comparable image quality associated with substantial radiation dose savings (effective dose: 0.4± 0.1 mSv vs. 2.0 ± 0.4 mSv) compared to a standard CTPA [10]. Unfortunately, the authors did not provide any information concerning patients’ size, weight or body-mass-index, so that the interpretation of the observed dose values is limited and the feasibility of their protocol regarding overweight or even obese patients that are increasingly more common in western clinical practice remains unclear. Lu et al assessed a high-pitch 80 kVp CTPA protocol with 20 ml contrast media in comparison to standard-pitch CTPA with 100 kVp and 60 ml contrast media, and observed a sufficient image quality of high-pitch 80 kVp CTPA in normal-weight individuals along with a significant reduction in radiation dose (effective dose: 0.9±0.2 mSv vs. 1.7±0.5 mSv) [11]. Even though Lu et al used only 20 ml contrast media for the CTPA scan, the total contrast media dosage was 30 ml in their study due to the fact that a test bolus of 10 ml contrast media was injected. Compared to these high-pitch, low kVp CTPA protocols our CTPA protocol has several advantages: First and foremost, we used standard-pitch acquisition which enabled the assessment of the ascending aorta comparably to standard CTPA even though only 25 ml of contrast media had been applied. Moreover, our protocol was not limited to normal-weighted individuals, which is important for the usability in daily routine.

The significant dose reduction in our study was achieved by decrease of the tube current and tube voltage [19, 20]. Moreover, lowering of the tube voltage leads to an increase in vascular attenuation due to an approximation to the K-edge of iodine. Besides these positive effects of lowering the tube voltage, a reduction of the tube voltage, especially in combination with lowering the tube current, results in an increased image noise. Therefore, we observed in our study slightly lower signal intensity, SNR and CNR values of the pulmonary artery in the low-dose low contrast media group. However, since we found a similar frequency of pulmonary embolism with both CTPA protocols and a sufficient subjective image quality with both CTPA protocols, the slightly lower objective image quality seems to be irrelevant in clinical practice.

Respiratory artifacts caused by dyspnea in patients with suspected pulmonary embolism can prove to be a potential disadvantage of standard pitch acquisition. However, our subjective image quality assessment showed a sufficient image quality of our standard-pitch protocols indicating that respiratory motion artifact had been no relevant problem in our study cohort. High-pitch acquisitions can increase image noise additionally, which may impair diagnostic capability especially for larger patients. Another limitation of an increased pitch is the reduction of scan time, which allows an excellent opacification of the pulmonary vasculature when an accurate delay is chosen. However, a simultaneous opacification of the ascending aorta is not possible when only small amount of contrast medium is injected [12]. Our investigational standard pitch protocol with 25 ml contrast agent showed an excellent or adequate image quality in 82% of all exams and allowed the exclusion of a dissection of the ascending aorta, which was not significantly inferior to our standard protocol (86%).

A limitation of our study is that we did not compare the two different CTPA protocols intraindividually, because it would have been unethical to perform more than one acquisition in each patient. As CTPA is the gold standard in clinical routine for the detection of PE and invasive pulmonary angiography is no longer performed in clinical routine, we were not able to verify the results regarding detection of pulmonary emboli.

## Conclusion

This study demonstrates that our 80 kVp standard pitch CTPA protocol with 25 ml contrast agent volume can obtain sufficient image quality to exclude or diagnose pulmonary emboli and dissection of the ascending aorta while reducing radiation dose by approximately 71%. This protocol is feasible for all patients without any limitations regarding patient size. It is especially beneficial for patients at risk for contrast-induced nephropathy or younger patients with an elevated risk for longterm-consequences of radiation exposure.
